# Compensatory movement detection by using near-infrared spectroscopy technology based on signal improvement method

**DOI:** 10.3389/fnins.2023.1153252

**Published:** 2023-05-10

**Authors:** Xiang Chen, YinJin Shao, LinFeng Zou, SiMin Tang, Zhiwei Lai, XiaoBo Sun, FaWen Xie, Longhan Xie, Jun Luo, Dongxia Hu

**Affiliations:** ^1^Department of Rehabilitation Medicine, The Second Affiliated Hospital of Nanchang University, Nanchang, China; ^2^Department of Rehabilitation Medicine, Ganzhou People's Hospital, Ganzhou, China; ^3^Ganzhou Hospital of Traditional Chinese Medicine, Ganzhou, China; ^4^Shien-Ming Wu School of Intelligent Engineering, South China University of Technology, Guangzhou, China

**Keywords:** compensatory movement detection, near-infrared spectroscopy, support vector machine, rehabilitation training, differential based signal improvement

## Abstract

**Introduction:**

Compensatory movements usually occur in stroke survivors with hemiplegia, which is detrimental to recovery. This paper proposes a compensatory movement detection method based on near-infrared spectroscopy (NIRS) technology and verifies its feasibility using a machine learning algorithm. We present a differential-based signal improvement (DBSI) method to enhance NIRS signal quality and discuss its effect on improving detection performance.

**Method:**

Ten healthy subjects and six stroke survivors performed three common rehabilitation training tasks while the activation of six trunk muscles was recorded using NIRS sensors. After data preprocessing, DBSI was applied to the NIRS signals, and two time-domain features (mean and variance) were extracted. An SVM algorithm was used to test the effect of the NIRS signal on compensatory behavior detection.

**Results:**

Classification results show that NIRS signals have good performance in compensatory detection, with accuracy rates of 97.76% in healthy subjects and 97.95% in stroke survivors. After using the DBSI method, the accuracy improved to 98.52% and 99.47%, respectively.

**Discussion:**

Compared with other compensatory motion detection methods, our proposed method based on NIRS technology has better classification performance. The study highlights the potential of NIRS technology for improving stroke rehabilitation and warrants further investigation.

## 1. Introduction

Stroke (Kuriakose and Xiao, 2020) is a leading cause of death and disability worldwide, with over 80% of stroke survivors suffer from upper limb motor function impairment with great pain (Hatem et al., 2016). Rehabilitation training is a proper way to recover, but it can be a large burden for stroke survivors. They need to adapt their movement patterns by recruiting trunk muscles and joints to compensate for the loss of motor function (Levin et al., 2009). While compensatory movement can significantly improve patient function in the short term, it hinders long-term recovery of motor function and reduces the effect of rehabilitation training (Cirstea and Levin, 2000). Therefore, detecting methods are needed to avoid compensatory movement.

Currently, the three most popular compensatory movement detection methods are body-worn technology, vision sensor technology, and motion capture systems (Wang et al., 2022). Inertial measurement units (IMUs) and accelerometers are commonly used in body-worn technology (Wang et al., 2017). They are placed on a patient's trunk, joint, shoulder, or other areas to monitor compensatory movements. But extra motions are needed to calibrate sensors which is quite a challenge for patients to perform these motions (Ranganathan et al., 2017). Vision sensor technology can picture patient motions through a camera or other vision sensor (Remoortel et al., 2012), and a motion capture system can obtain more accurate posture information with markers placed on key parts of the body (Bakhti et al., 2018). Both are non-contact technologies that remove the constraints of attached sensors (Taati et al., 2012; Subramanian et al., 2013). However, they both require a well-illuminated environment to obtain reliable results, and patients are more resistant because of privacy issues (Mündermann et al., 2006). Moreover, there are other compensatory movement detection technologies. A pressure distribution mattress is a simple and low-cost method that can capture a patient's sitting posture since different compensatory movements lead to different sitting postures (Cai et al., 2019b).

Nevertheless, the above methods all face the same problem. They cannot provide appropriate guidance on how to avoid compensatory movements. A better choice is to use physiological signals, which can directly reflect muscle functions. Surface electromyography (sEMG) signal (Vigotsky et al., 2018) and Near-infrared spectroscopy (NIRS) signals (Ferrari and Quaresima, 2012; Scholkmann et al., 2014) are two commonly used physiological signals. They both can detect the degree of activity of muscles so therapists can better understand the patterns in which compensatory movement occurs and flexibly guide patients to correct them (Yang et al., 2015). Specifically, the sEMG signal is an electrical signal that reflects a bioelectrical change during muscle activation (Kauppi et al., 2017), while the NIRS signal is an optical signal that reflects the hemodynamic change during muscle activation. Compared with sEMG, NIRS is cleaner with better resistance to electromagnetic interference. And NIRS signal contains two types of signals, oxyhemoglobin (*Hb*) and deoxyhemoglobin (*HbO*_2_), so it can reflect more information on muscle states. For patients, Optical signals are more receptive than electrical signals. Moreover, the NIRS signal has higher spatial resolution (Guo et al., 2016).

The results of the sEMG signal in detecting compensatory movement have proven its superior performance, demonstrating that physiological signal is better than other signals (Ma et al., 2019). NIRS signal has also been adapted to trunk and limb motion detection, and the results showed its effectiveness (Guo et al., 2017; Sheng et al., 2021; Stöggl and Born, 2021). However, no research has evaluated the validity of NIRS signals in detecting compensatory movement. Therefore, this study aims to validate the effectiveness of the NIRS signals in detecting compensatory movements.

## 2. Methods

### 2.1. Participants

Ten healthy subjects (All Male, Age in 24 ± 2.3 years, with no upper limb dysfunction) and six stroke survivors with different degrees of upper limb function impairment were invited to participate in this study. The details of the stroke survivors were listed in Table 1. Stroke survivors were recruited during their in-patient hospitalization at the Second Affiliated Hospital of Nanchang University. Recruitment was done under the guidance of an experienced rehabilitation therapist. This study was approved by the Ethics Committee of the Second Hospital of Nanchang University, with all the participants providing informed consent. All the research was performed in accordance with the Declaration of Helsinki.

**Table 1:** Details of the stroke survivors.

**Subjects**	**Sex**	**Age**	**BS^*a*^**	**AS^*b*^**	**Month^*c*^**
S1	M	56	III	Left	4
S2	F	67	IV	Left	1
S3	M	40	IV	Left	3
S4	F	67	III	Left	12
S5	M	56	IV	Left	1
S6	M	37	IV	Left	1

^*a*^BS, Brunnstrom scale. ^*b*^AS, affected side. ^*c*^Months, time since first stroke.

The recruitment of participants follows the following criteria: (1) Ages between 18 and 70 years; (2) within 1 year since the first stroke. (3) the Brunnstrom Scale above level II, with upper limb exercise ability. (4) have a good level of cognition and can understand simple instructions. (5) the ability to maintain a sitting position (6) with no obvious spasticity.

### 2.2. Experimental setup

**Figure 1:**
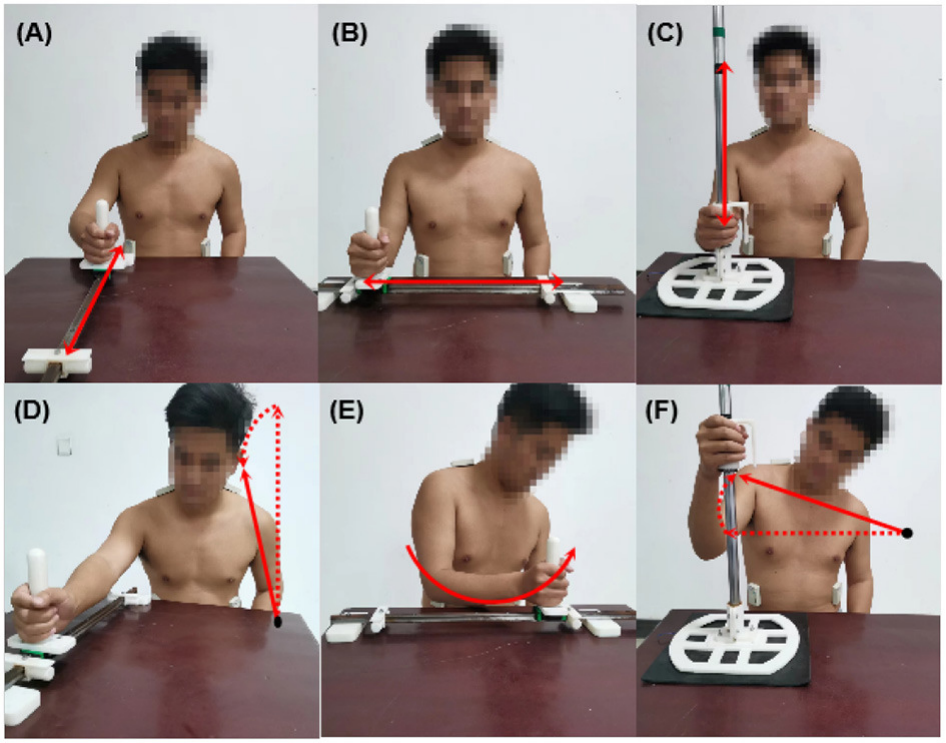
Three types of rehabilitation training motions and corresponding compensatory movements. **(A)** Reach-forward-to back (FB); **(B)** reach-left-to-right (LR); **(C)** reach-up-to-down (UD); **(D)** lean forward (LF); **(E)** trunk rotation (TR); **(F)** shoulder elevation (SE).

In this experiment, participants were asked to perform three basic rehabilitation training motions: reach-forward-to-back (FB), reach-left-to-right (LR), and reach up-to-down (UD), as shown in Figures 1A–C. These three motions can provide a good exhibition of a participant's upper limb function. Compensatory movements will happen when participants try to accomplish these movements. Specifically, the FB motion will compel the participant to lean their trunk forward, which leads to lean forward (LF) compensation, as shown in Figure 1D; the LR motion will compel the participant to rotate their trunk, which leads to trunk rotation (TR) compensation, as shown in Figure 1E; and the UD motion will compel a participant to elevate their shoulder, which leads to shoulder elevation (SE) compensation, as shown in Figure 1F.

#### 2.2.1. NIRS acquisition system

**Figure 2:**
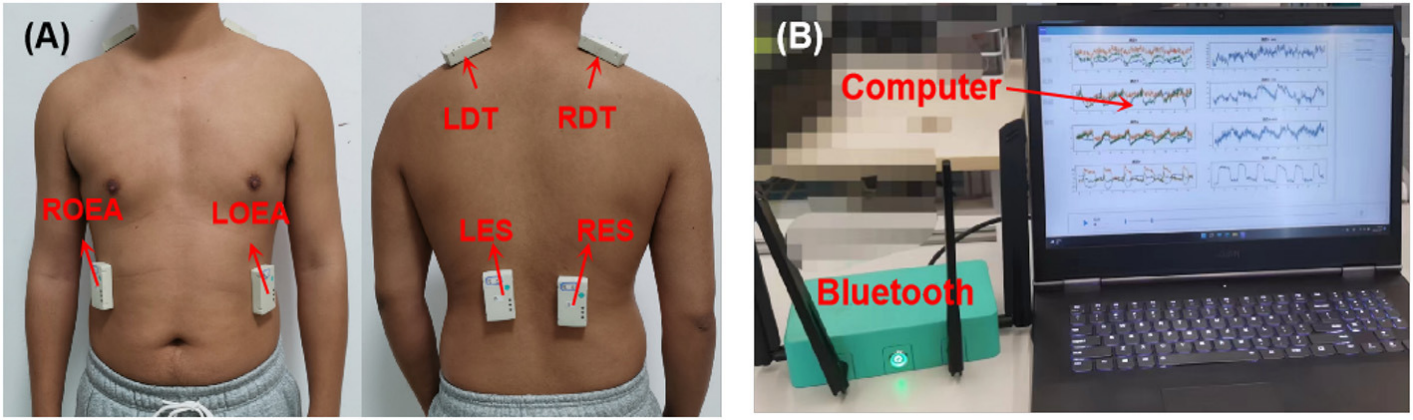
The experimental setup. **(A)** The placement of NIRS sensors on the trunk muscles. **(B)** NIRS acquisition system, including Bluetooth and upper computer.

Many muscles in the trunk are involved in movements, and with the help of therapists, six muscles were selected to detect compensatory movements, which are the left and right obliquus externus abdominis (LOEA and ROEA), the left and right descending part of trapezius muscles (LDT and RDT), and the left and right erector spinae (LES and RES), as shown in Figure 2A.

During rehabilitation training, the activated situation of the six muscles was acquired through a wireless multichannel NIRS system. The wireless NIRS system has three different wavelengths of near-infrared light (750, 800, and 850 nm) and dual photodiodes to offer a high precision of light intensity changes. An ambient light cancellation circuit prevents ambient light interference to guarantee the quality of the NIRS signal. Meanwhile, all the NIRS data were transmitted to an upper computer through Bluetooth and displayed in real-time, as shown in Figure 2B. The sampling frequency of this NIRS system is 16 Hz. More detailed information can be found in Xie et al. (2022).

#### 2.2.2. Data collection

Since it is the first time that the NIRS signal has been used in compensatory movement detection, before we performed the experiments on stroke survivors, we first performed simulated compensatory experiments on healthy subjects (Zhi et al., 2017). To simulate the compensatory movement of an actual stroke survivor, an orthotic was used to restrict the range of motion of each subject's upper limbs, and all motions were completed under the guidance of researchers.

The NIRS sensors were placed on the participant's body over the muscles mentioned above and secured with tape to prevent them from slipping. Wait a few minutes and let the sensors adjust the body temperature to reduce the influence of temperature drift. Afterward, each participant was required to sit in front of a table on an adjustable chair with no back or arm, which might restrict the trunk and limb movement. The chair was adjusted to let the participant lay their legs on the ground. Under the guidance of a therapist, the subjects performed the three training motions (FB, LR, and UD) using their lateral hand to simulate compensation motions with the assistance of a guideway and handle, as in Figure 1. These motions correspond to the three different kinds of compensation: LF, TR, and SE. Each participant was required to perform the three motions 20 times each. They could rest for 10 s after completing one motion and 2 min after finishing 10 motions to avoid muscle fatigue. In addition, subjects were required to perform each motion ten times with no simulation, and these data were marked as no compensatory (NC). As for stroke survivors, they performed the three motions using their affected hands, the data was regarded as compensatory movements, and the no compensatory movement data was collected using the healthy hands. The NIRS system recorded the muscle activation data of all subjects, which were used for further data processing.

### 2.3. Data processing

After data acquisition, all the data were processed using Python (version 3.8, Python Software Foundation, Hampton, NH), including data preprocessing, signal improvement, feature extraction, and classification. Moreover, a sliding window with a window size of 500 ms (eight samples) and stride size of 250 ms (four samples) was used to extract time-domain features.

#### 2.3.1. Data preprocessing

In the process of wireless transmission, some values may be missed due to various interferences. Therefore, the average of four points before a missing point was chosen as a padding value. After that, a sixth-order Butterworth lowpass filter with a 0.5 Hz cutoff frequency was used to eliminate the interference of high-frequency noise and heart rate. EMD decomposition was used to eliminate the baseline drift of the raw NIRS signal.

#### 2.3.2. NIRS signal improvement

As mentioned, the NIRS data contain two signals: *Hb* and *HbO*_2_. The contraction of muscles causes different changes in *Hb* and *HbO*_2_. Cui et al. (2010) found that *Hb* is negatively correlated with *HbO*_2_ and proposed a correlation-based signal improvement (CBSI) method to improve the signal quality. The CBSI method assumes that *Hb* should be perfectly negatively correlated to *HbO*_2_ and ignores the identical effects on *Hb* and *HbO*_2_. The CBSI method is calculated as follow:


(1) $$\begin{array}{l} \{\begin{array}{c} \alpha =\frac{std\left(HbO_{2}\right)}{std\left(Hb\right)}\\ TNS=\frac{1}{2}\left(HbO_{2}+\alpha *Hb\right)\\ TFS=\frac{1}{2}\left(HbO_{2}-\alpha *Hb\right) \end{array} \end{array}$$

where α is a constant positive factor, the true noise signal (TNS) refers to the identical effects on *Hb* and *HbO*_2_, and the true functional signal (TFS) refers to the actual functional components that correlate to the motions.

In fact, the standard CBSI method only focuses on the TFS, and the TNS is considered as noise. The above function is used to maintain consistency with the DBSI method mentioned later.

However, there are two factors that cause *Hb* and *HbO*_2_ concentration changes: blood flow and oxygen consumption. The former leads to a positive correlation between *Hb* and *HbO*_2_, while the latter causes a negative correlation (Xie et al., 2022). Therefore, a differential-based signal improvement (DBSI) method is proposed.


(2) $$\begin{array}{l} \{\begin{array}{c} CMS=\frac{1}{2}\left(HbO_{2}+Hb\right)\\ DMS=\frac{1}{2}\left(HbO_{2}-Hb\right) \end{array} \end{array}$$

where the common mode signal (CMS) refers to the common component, which represents the blood flow. The differential mode signal (DMS) refers to the differential component representing oxygen consumption.

**Figure 3:**
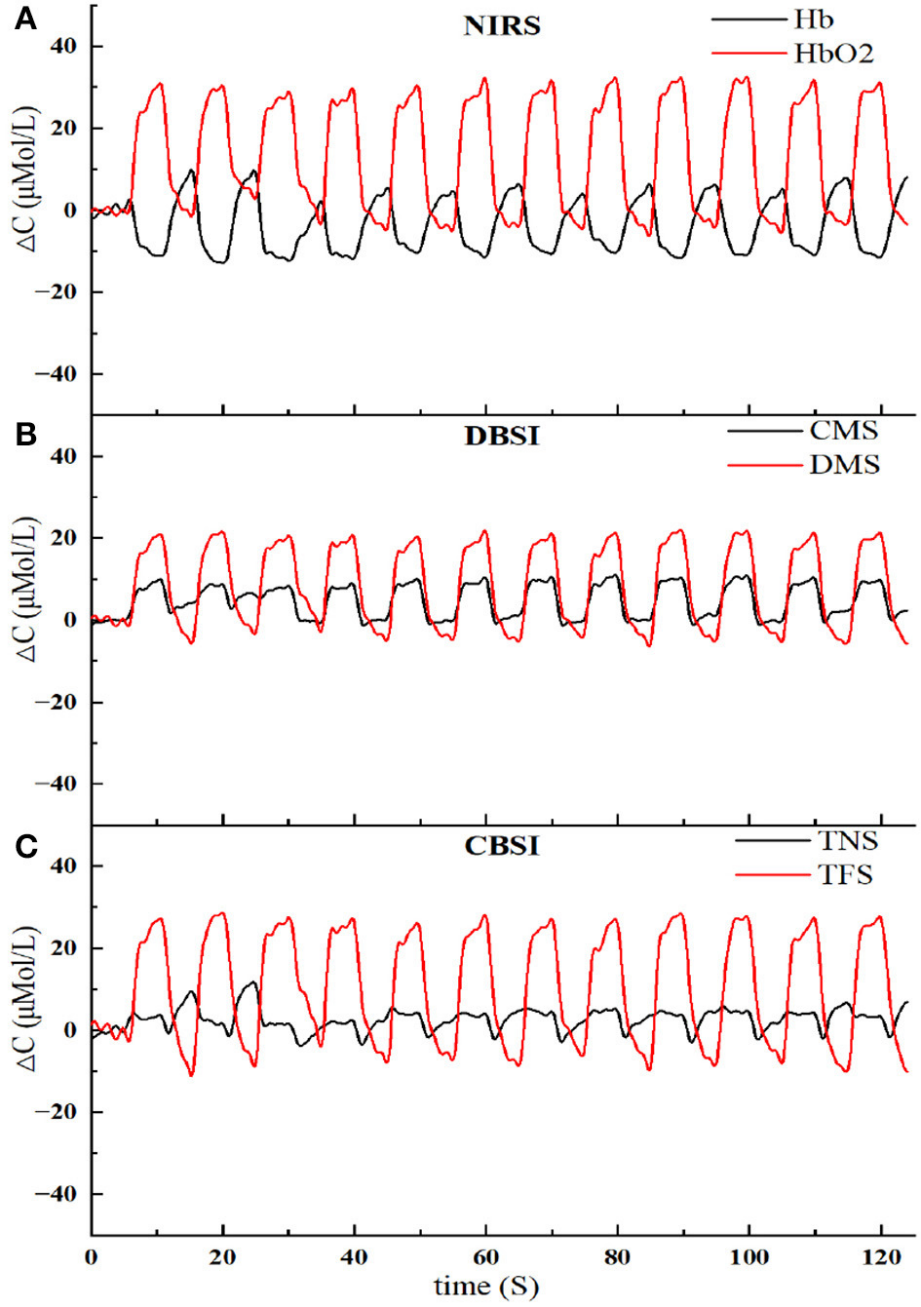
The results of NIRS signal conversion using two methods. **(A)** The original NIRS signal, including *Hb* and *HbO*_2_. **(B)** Signal conversion based on the DBSI method, including CMS and DMS. **(C)** Signal conversion based on the CBSI method, including TNS and TFS.

To analyze the effectiveness of the two signal conversion modes, both methods were used to convert the original NIRS signal, and the results are shown in Figure 3.

#### 2.3.3. Feature extraction

When a muscle is activated or deactivated, it will take a few seconds for the hemoglobin concentration to increase or decrease and reach a new plateau. Meanwhile, the concentration change rate has been positively correlated with the degree of muscle activation. The linear fitting slope (LFS) was used to estimate the muscle activation segment.


(3) $$\begin{array}{l} LFS=\frac{\overset{k}{\underset{i=1}{\sum }}\left(x_{i}*\left(x_{i}-\bar{t}\right)\right)}{\overset{k}{\underset{i=1}{\sum }}x_{i}^{2}-{\bar{t}}^{2}} \end{array}$$

where k is the window size, *x*_*i*_ is the NIRS signal, and $\bar{t}$ is the mean time value at the analysis window.

The NIRS signal segments can be divided into *n* segments by the sliding window, and the LFS value $LFS_{m}^{n}$ can be calculated from each small segment. Calculated the difference between $LFS_{m}^{n}$ for one window-sized segment and *LFS*_*m*_ for the whole signal. If the mean value of $LFS_{m}^{n}$ is approximately equal to *LFS*_*m*_ and not approximately equal to zero, then it can be considered an active segment. *LFS*_*m*_ represents the muscle activation degree. The deactivation segment detection is the same process.

Since the power spectrum of the NIRS signal is mainly concentrated in the low-frequency band and the sampling frequency is not high (Pinti et al., 2019), it is better to select time domain features rather than frequency domain features. After comparing and analyzing different time domain features and considering the characteristics of the NIRS signal, two commonly used time domain features are selected: the mean value (MEA) and standard deviation (STD) of the NIRS sensors. The MEA can reflect the relative hemoglobin concentration change, and the STD reflects the stationary hemoglobin concentration.


(4) $$\begin{array}{l} \{\begin{array}{c} MEA=\frac{1}{k}\overset{k}{\underset{i=1}{\sum }}x_{i}\\ STD=\frac{1}{k}\overset{k}{\underset{i=1}{\sum }}{\left(x_{i}-MEA\right)}^{2} \end{array} \end{array}$$

#### 2.3.4. Classification

To comprehensively evaluate the performance of the NIRS signal in compensatory movement detection, a support vector machine (SVM) was used for classification (Cortes and Vapnik, 1995). SVM is a widely used classification algorithm that constructs a hyperplane to maximize different labels. Since the hyperplane is only related to the support vectors on the boundary, SVM has a very small model size and high stability, which is suitable for small sample detection. Moreover, the introduction of a kernel function enables an SVM to address high-dimensional data. The segmentation principle is to maximize the interval and finally transform it into a convex quadratic programming problem (Noble, 2006), expressed as:


(5) $$\begin{array}{l} \{\begin{array}{c} \min\frac{1}{2}∥\omega {∥}^{2}\\ s.t.y_{i}\left(\omega^{T}x_{i}+b\right)-1\geq 0,\;i=1,2,\ldots ,N \end{array} \end{array}$$

where ***x*_*i*_** is the feature vector, *y*_*i*_ is the sample label, and (**ω**, *b*) is the hyperplane parameter.

Since SVM can only realize a two-label classification, a one-versus-one strategy (Hsu and Lin, 2002) was used for this four-label classification (LF, TR, SE, NC), which was achieved through the LIBSVM package (Chang and Lin, 2011). The Gaussian radial basis function (RBF) was used as the kernel function, and the penalty factor C was set as 0.3.

At last, the dataset size of healthy subjects was 11,505, and the dataset size of stroke survivors was 5,652. The experiment has six NIRS sensors, each sensor can obtain two signals: *Hb* and *HbO*_2_, and each signals can extract two features, so the samples have 24 features. Tenfold cross-validation was used to assess classifier performance. After normalization, all the data were randomly and equally divided into ten parts. For each iteration, one of the ten parts was selected with no repetition as the testing dataset, and the rest were used as the training dataset. The final result was the average accuracy of the ten times cross-validation. Finally, a confusion matrix was used to evaluate the performance of the model by intuitively showing the relationship between the predicted and true values.

## 3. Results

**Figure 4:**
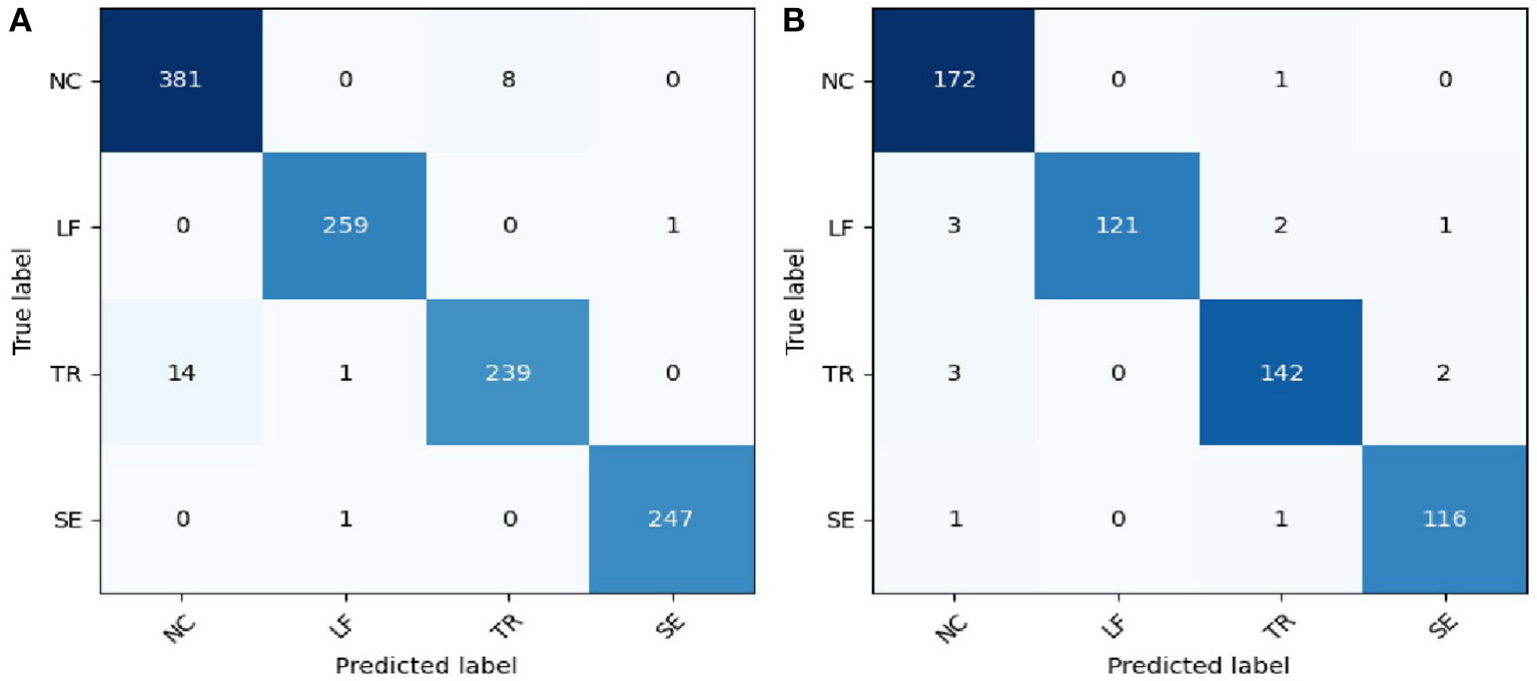
Confusion matrix of the compensatory movement detection based on NIRS signal. **(A)** Healthy participants; **(B)** stroke survivors. NC, no compensatory; LF, lean-forward; TR, trunk rotation; SE, shoulder elevation.

The results of the SVM classification based on the NIRS signal in detecting compensatory movement were evaluated using a confusion matrix, as shown in Figure 4. The confusion matrix represents the classification results for healthy subjects and stroke survivors. For healthy subjects, the classification accuracy for this validation was 97.74%, with the majority of misclassified samples belonging to the NC and TR categories. Similarly, for stroke survivors, the classification accuracy for this validation was 97.52%, with only a small number of misclassified samples in each category.

**Figure 5:**
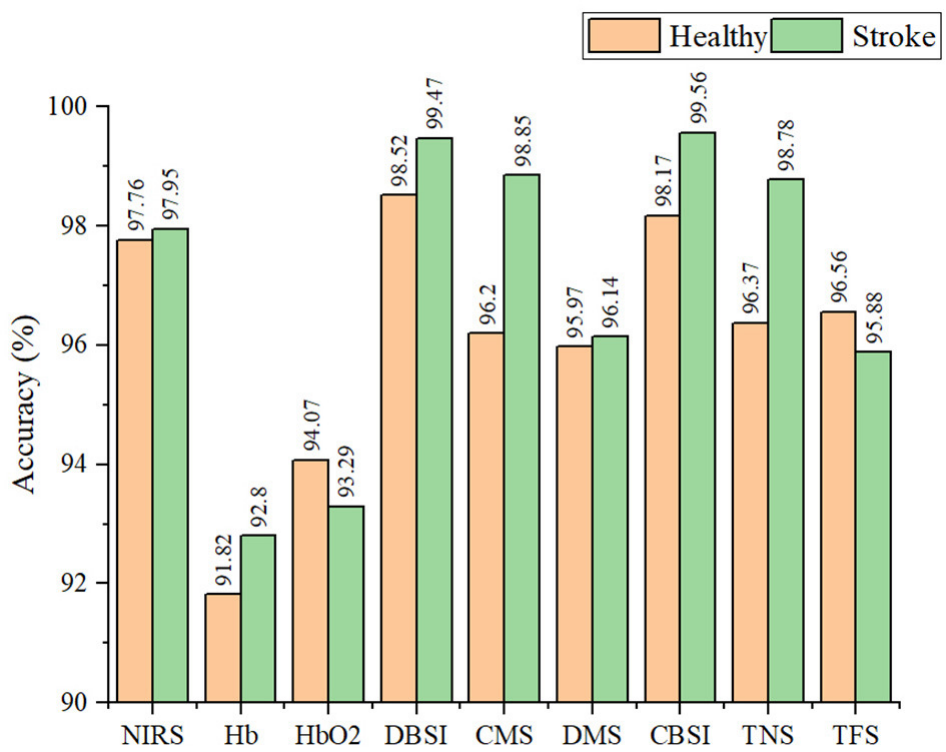
The classification accuracy of the SVM classifier in detecting compensatory movement by using different types of signals on healthy and stroke subjects.

The classification performance of the SVM classifier in detecting compensatory movements by using different types of signals on healthy subjects and stroke survivors was shown in Figure 5. The results showed good performance, with the classification accuracy for healthy subjects (97.76%) slightly lower than that for stroke survivors (97.95%) based on the original NIRS signal. After applying signal improvement methods, the classification accuracy for both healthy and stroke subjects was improved, with the accuracy for healthy subjects (98.52%) still lower than that for stroke survivors (99.56%). Comparing the two signal improvement methods, CBSI and DBSI, it can be found that the effects of the two were similar. However, compared with the standard CBSI method, namely the TFS signal, the DBSI method (99.47%) was significantly better than TFS (95.88%). When comparing the two components of the DBSI signal, the accuracy of CMS (98.85%) was higher than that of DMS (96.14%).

Precision, recall, and F1-score are indicators used to measure the classification performance of the SVM classifier on four types of compensatory categories, including NC, LF, TR, and SE. Precision refers to how many predicted samples are accurate; Recall refers to how many true samples are predicted correctly; the F1 score is a comprehensive index, which is the harmonic mean of precision and recall. Table 2 listed the performance of the SVM classifier in recognizing the four categories of healthy and stroke subjects using DBSI signals. For healthy subjects, the best accuracy performance was achieved on the LF label (F1-score = 99.79%), followed by SE (F1-score = 99.66%), NC (F1-score = 97.95%), and TR (F1-score = 96.83%). For stroke survivors, NC, LF, and TR labels obtained excellent classification performance with F1-score higher than 99.5%, and the F1-score of the SE label was 98.87%.

**Table 2:** Classification performance of the SVM classifier in recognizing four different compensatory categories of healthy and stroke subjects using the DBSI method.

		**NC**	**LF**	**TR**	**SE**
Healthy	Precision	98.64	99.57	95.66	99.96
	Recall	97.28	100	98.06	99.37
	F1-score	97.95	99.79	96.83	99.66
Stroke	Precision	99.72	99.25	99.64	99.17
	Recall	99.88	99.77	99.48	98.58
	F1-score	99.80	99.51	99.56	98.87

**Figure 6:**
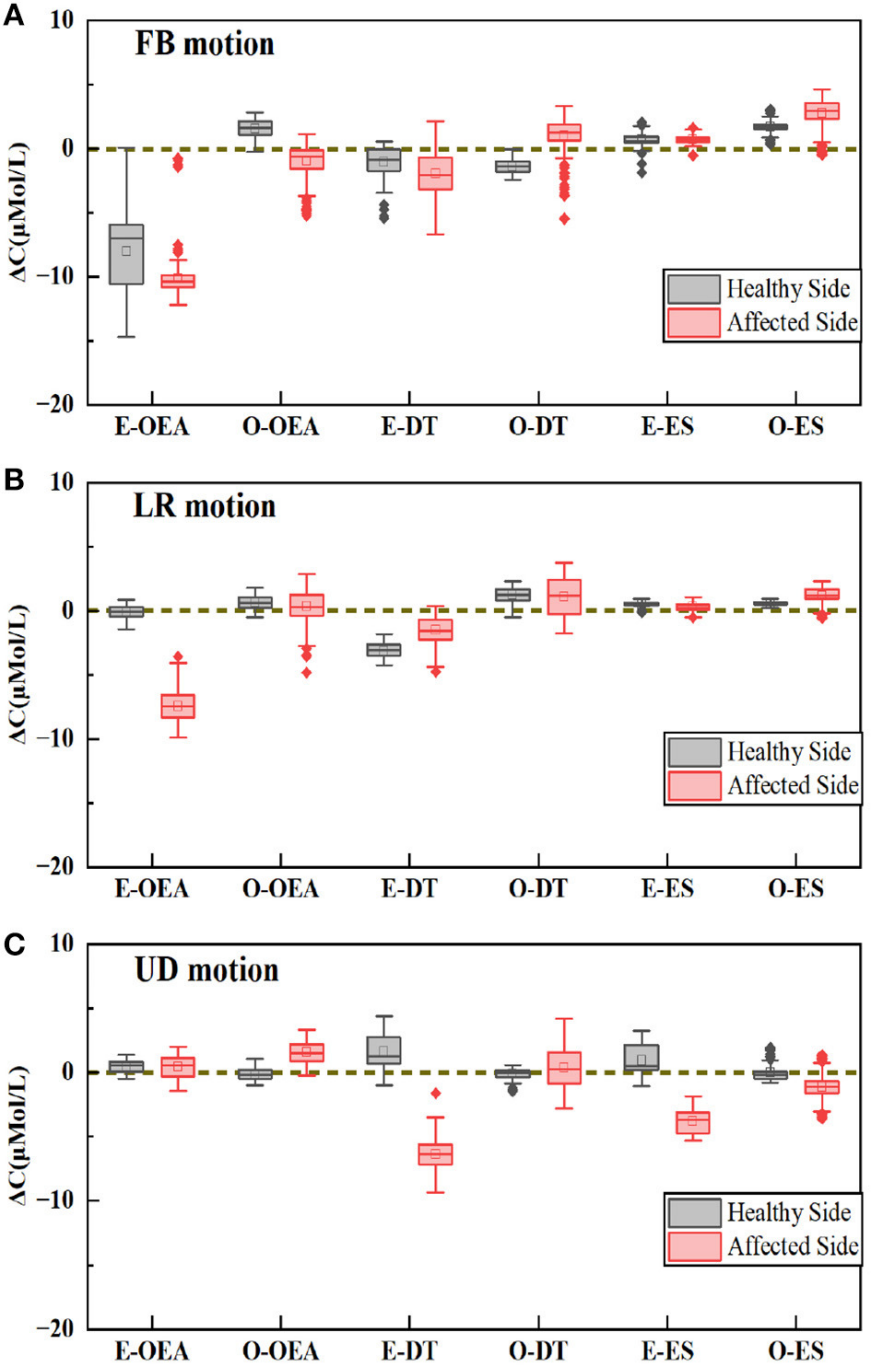
The mean value of CMS signal on six muscles, including two OEAs, two ESs, and two DTs. **(A)** The forward and back (FB) motion. **(B)** The left and right (LR) motion. **(C)** The up and down (UD) motion.

Based on the superior classification accuracy of the CMS signal shown in Figure 5, the mean value of the CMS signal activation segment in the six muscles for stroke survivors during three reaching tasks using the healthy and affected hand was calculated and depicted in the box diagram, as shown in Figure 6. The prefix E and O in the abscissa indicate the executing and opposite sides related to the executing hand. For the affected hand, the executing side was the left hand, and the opposite was the right. For the healthy side, the executive side was the right hand, and the opposite side was the left hand. The size of the box in the activation signal box plot represents the dispersion of the signal, and it was found to be smaller when using the healthy hand for stroke survivors, indicating that they were able to complete the tasks more easily using their healthy hand. The distance of the box from the baseline of 0 represents the intensity of the activation signal, with a greater distance indicating a higher level of activation.

In the case of the FB motion, the primary compensatory muscle was found to be E-OEA for both the affected and healthy hands, while O-OEA and O-DT showed different performances, as shown in Figure 6A. In the case of the LR motion, the primary compensatory muscle was E-OEA, with E-DT showing different performances, as shown in Figure 6B. For the UD motion, the primary compensatory muscles were E-DT and E-ES, while O-ES showed different performances, as shown in Figure 6C.

## 4. Discussion

In this paper, we propose a novel method for detecting compensatory movements based on NIRS signals, using six trunk muscles to verify the type of compensatory pattern. The NIRS signal contains two components, *Hb* and *HbO*_2_, which reflect the changes in oxygen content during muscle contraction. Two commonly used time-domain features: mean value and variance, were extracted from the active segments of each channel and each component. An SVM algorithm was used for a four-classification model, including no compensatory (NC) movement and three simulated compensatory movements (LF, TR, and SE). Ten healthy subjects and six stroke survivors were recruited to train and test the model separately, which achieved good classification performance. Furthermore, a differential-based signal improvement (DBSI) method was proposed to enhance the performance of this model.

Table 3 presents the detection performance of different compensatory movement detection methods. Although several factors can affect the detection result, such as the differences in subjects and models used, the NIRS method proposed in this study achieved the highest detection performance in both healthy subjects and stroke survivors, with F1-scores of 98.5 and 99.4%, respectively. This indicates the effectiveness of the NIRS technique in compensatory movement detection. In addition, in the pressure and NIRS method, the detection precision in stroke survivors was higher than in healthy subjects, while it was the opposite in the sEMG method. This may be due to the different BS level among stroke survivors. Subjects with a higher BS level could perform the reaching tasks. As for the subjects with lower BS level, they would produce a more complex compensatory behavior, which reduced the detection performance in stroke survivors. Conversely, for healthy subjects, the detection accuracy was related to the quality of their simulation compensatory movement, which cannot perfectly mimic real stroke survivors, leading to reduced detection performance in healthy subjects.

**Table 3:** The compensatory detection performance among different methods.

**Method**	**Model**	**F1-score**	
		**Healthy (%)**	**Stroke (%)**
Accelerometer (Ranganathan et al., 2017)	Byers	88.6	–
Vision (Lin et al., 2021)	1D-CNN	–	97.5
Pressure (Cai et al., 2019a,b)	SVM	90.5	98.1
sEMG (Ma et al., 2019; Chen et al., 2021)	SVM	95.5	95
NIRS	SVM	98.5	99.4

During muscle activation, the NIRS signal was primarily influenced by the changes of blood flow and oxygen consumption. The signal improvement method proposed in this paper, DBSI, could separate these two factors from the original NIRS signal, leading to improved signal quality and a 1.5% increase in detection performance. Further analysis revealed that blood flow were more sensitive than oxygen consumption in compensatory detection, as shown in Figure 5. On the other hand, the CBSI method is commonly used in fNIRS signal analysis, which mainly focuses on oxygen consumption and treats blood flow changes as noise. Therefore, the DBSI method is more suitable for muscle NIRS signal detection, providing a more comprehensive understanding of muscle state changes.

As a direct detection method, the NIRS technique could provide a specific understanding of which muscles were involved in the compensatory movement and clear guidance to therapists and patients on how to avoid compensatory. As shown in Figure 6, E-OEA was mainly activated in FB and LF motions; E-DT and E-ES were mainly activated during UD action. The three opposite side muscles, O-OEA, O-DT, and O-ES, were slightly activated during the arrival motions, indicating that the opposite side muscles also played a certain auxiliary role in compensatory movement detection.

This study also has some limitations. The number of subjects recruited in this study was insufficient, and their BS level was relatively good, which might be the factor that resulted in the high measurement accuracy. The arrival tasks and compensation mode in this paper were also relatively simple. In the actual scenario, the compensatory pattern of stroke survivors could be more complex, which puts higher requirements on detection techniques and methods. In order to better adapt to compensatory detection in real scenarios, more stroke survivors with different BS levels will be recruited in future work. What's more, a compensatory detection model with a better detection effect and higher accuracy will be built. At the same time, a multi-source data fusion approach, such as combining NIRS with sEMG, can also be tried to combine the advantages of different types of signals to improve classification performance.

## 5. Conclusion

The main purpose of this study was to develop a simple, effective, and accurate compensatory movement detection method. NIRS technology can detect changes in near-infrared light attenuation to determine the state of muscles. An accurate and effective compensatory movement detection method can be achieved by monitoring specific muscles in real time, which can help subjects realize better guidance on how to avoid compensatory movements. A differential-based signal improvement method was applied to improve the signal quality. An SVM algorithm was used to classify and predict the no compensatory (NC) movement and three commonly used compensatory movements, including lean-forward (LF), trunk rotation (TR), and shoulder elevation (SE), of healthy subjects and stroke survivors. The experimental results showed that the NIRS signal was superior to other methods in compensation movement detection, and its classification accuracy reached 98.52% in healthy subjects and 99.47% in stroke survivors. In future studies, we will continue to explore the effects of NIRS techniques on compensatory movement detection in stroke survivors suffering from hemiplegia and optimize the methods to accommodate more complex true compensatory behavior.

## Data availability statement

The original contributions presented in the study are included in the article/supplementary material, further inquiries can be directed to the corresponding authors.

## Ethics statement

The studies involving human participants were reviewed and approved by Biomedical Research Ethics Committee of the Second Affiliated Hospital of Nanchang University. The patients/participants provided their written informed consent to participate in this study. Written informed consent was obtained from the individual(s) for the publication of any potentially identifiable images or data included in this article.

## Author contributions

XC and YS designed the research and participated in the entire research including data collection, data processing, model construction, result interpretation, manuscript drafting, and revisions. DH and JL designed the research and participated in the data collection and revisions of the manuscripts. LZ and LX designed the research, manuscript drafting, and revisions. ZL and XS participated in the data collection. ST and FX participated in the analysis of the results. All authors contributed to the article and approved the submitted version.
